# TMEM97 and PGRMC1 do not mediate sigma-2 ligand-induced cell death

**DOI:** 10.1038/s41420-019-0141-2

**Published:** 2019-01-28

**Authors:** Chenbo Zeng, Chi-Chang Weng, Mark E. Schneider, Laura Puentes, Aladdin Riad, Kuiying Xu, Mehran Makvandi, Linda Jin, William G. Hawkins, Robert H. Mach

**Affiliations:** 1 Department of Radiology, Perelman School of Medicine, University of Pennsylvania, Room 283 231S. 34th St, Philadelphia, PA 19104 USA; 2 Department of Surgery, Washington University School of Medicine, St. Louis, MO 63110 USA

## Abstract

Sigma-2 receptors have been implicated in both tumor proliferation and neurodegenerative diseases. Recently the sigma-2 receptor was identified as transmembrane protein 97 (TMEM97). Progesterone receptor membrane component 1 (PGRMC1) was also recently reported to form a complex with TMEM97 and the low density lipoprotein (LDL) receptor, and this trimeric complex is responsible for the rapid internalization of LDL. Sigma-2 receptor ligands with various structures have been shown to induce cell death in cancer cells. In the current study, we examined the role of TMEM97 and PGRMC1 in mediating sigma-2 ligand-induced cell death. Cell viability and caspase-3 assays were performed in control, TMEM97 knockout (KO), PGRMC1 KO, and TMEM97/PGRMC1 double KO cell lines treated with several sigma-2 ligands. The data showed that knockout of TMEM97, PGRMC1, or both did not affect the concentrations of sigma-2 ligands that induced 50% of cell death (EC_50_), suggesting that cytotoxic effects of these compounds are not mediated by TMEM97 or PGRMC1. Sigma-1 receptor ligands, (+)-pentazocine and NE-100, did not block sigma-2 ligand cytotoxicity, suggesting that sigma-1 receptor was not responsible for sigma-2 ligand cytotoxicity. We also examined whether the alternative, residual binding site (RBS) of 1,3-Di-*o*-tolylguanidine (DTG) could be responsible for sigma-2 ligand cytotoxicity. Our data showed that the binding affinities (*K*_i_) of sigma-2 ligands on the DTG RBS did not correlate with the cytotoxicity potency (EC_50_) of these ligands, suggesting that the DTG RBS was not fully responsible for sigma-2 ligand cytotoxicity. In addition, we showed that knocking out TMEM97, PGRMC1, or both reduced the initial internalization rate of a sigma-2 fluorescent ligand, **SW120**. However, concentrations of internalized **SW120** became identical later in the control and knockout cells. These data suggest that the initial internalization process of sigma-2 ligands does not appear to mediate the cell-killing effect of sigma-2 ligands. In summary, we have provided evidence that sigma-2 receptor/TMEM97 and PGRMC1 do not mediate sigma-2 ligand cytotoxicity. Our work will facilitate elucidating mechanisms of sigma-2 ligand cytotoxicity.

## Introduction

The sigma receptor was originally defined pharmacologically^1^. The sigma receptor was once thought to be a subset of the opioid receptor^2^, but was subsequently revealed to be a distinct class of receptors^1^. There are two subtypes of sigma receptors, sigma-1 and sigma-2 receptors. The molecular weight of sigma-1 and sigma-2 receptors was previously reported to be 25 and 18–21.5 kD, respectively^3^. The sigma-2 receptor has been shown to be expressed in higher density in proliferating versus quiescent tumor cells^4,5^. Sigma-2 ligands have been developed as molecular probes for imaging solid tumors and also as potential therapeutic agents for treating cancer^6^. A sigma-2 receptor ligand has also shown promise as a novel approach for treating Alzheimer’s disease (AD)^7,8^.

Recently, transmembrane protein 97 (TMEM97), a protein implicated in cancer and cholesterol homeostasis, was identified as the sigma-2 receptor^9^. TMEM97 has a role in cholesterol and lipid metabolism^10–12^, and it was reported that TMEM97 and cholesterol biosynthesis genes in normal ovarian epithelial cells were coordinately upregulated by progesterone treatment^10^. In another report, TMEM97 was identified as a regulator of cellular cholesterol homeostasis in targeted RNAi screening^11^. TMEM97 knockdown reduced cellular cholesterol levels as well as the rate of internalization of low density lipoprotein (LDL) uptake by the LDL receptor (LDLR). Furthermore, cell cultures growing under sterol-depleted conditions results in an upregulation of TMEM97 mRNA levels^11^. These data indicated that TMEM97 has a key role in the regulation of cholesterol homeostasis.

Previously, our group proposed that the binding site for the sigma-2 receptor resided in a protein complex containing the progesterone receptor membrane component 1 (PGRMC1)^13^. Subsequent studies challenged the validity of our results^14,15^, and the recent identification of the TMEM97 as the gene for the sigma-2 receptor seemed to substantiate these reports^9^. However, our group recently reported that TMEM97 formed a complex with PGRMC1 and LDLR, and this trimeric complex was responsible for the rapid internalization of LDL in HeLa cells^16^. These data demonstrated that PGRMC1 associates with TMEM97 physically and functionally, and corroborates our previous report that the sigma-2 receptor represents a binding site in the PGRMC1 protein complex.

TMEM97 (initially called MAC30) was first reported as a differentially expressed gene in meningioma^17^. TMEM97 was decreased in meningioma compared to normal leptomeningeal tissues. Subsequent studies showed that TMEM97 was overexpressed in gastric cancer^18^, colorectal cancer^19^, breast cancer^20^, glioma^21^, and ovarian cancer^22^ cells. Overexpression of TMEM97 was positively correlated with tumor stage, metastasis, and shorter survival time of patients with various cancers^19,22^. The differential expression of TMEM97 in cancer cells suggest that this protein may have a role in tumor development, growth, and proliferation.

The sigma-2 receptor is a potential target for cancer therapeutics^6,23^. Sigma-2 ligands have been shown to induce cytotoxicity in cancer cells as a single agent, a drug delivery agent^24,25^ and a sensitizer to other anticancer drugs in cell culture and in animal models. Sigma-2 ligands trigger cell death by inducing lysosome dysfunction, ROS production, caspase-independent, and caspase-dependent events^26–31^. However, it is not known how sigma-2 ligands interact with sigma-2 receptors and induce subsequent cell death. Sigma-2 ligands have nanomolar binding affinities to sigma-2 receptors but require micromolar range of concentrations to exhibit cell-killing effects^6^. The factors responsible for the large difference between sigma-2 receptor affinity and potency in the cell-kill assays are currently unknown.

In the current study, we studied whether TMEM97 or PGRMC1 mediated sigma-2 ligand-induced cell death. We show that CRISPR/Cas9 gene editing of TMEM97 and PGRMC1 did not affect the cytotoxicity of a panel of structurally diverse sigma-2 ligands. We also examined whether the alternative residual binding site of DTG, a gold standard sigma-2 ligand, in TMEM97 knockout (KO) and TMEM97/PGRMC1 double KO cells could be responsible for sigma-2 ligand cytotoxicity. Our data showed that this residual binding site of DTG does not appear to be responsible for the cytotoxicity of sigma-2 ligands, and raises a question regarding the mechanism of action of these ligands as anticancer drugs.

## Results

### Knockout of TMEM97, PGRMC1, or both proteins did not affect EC_50_ values of sigma-2 ligands

**Fig. 1:**
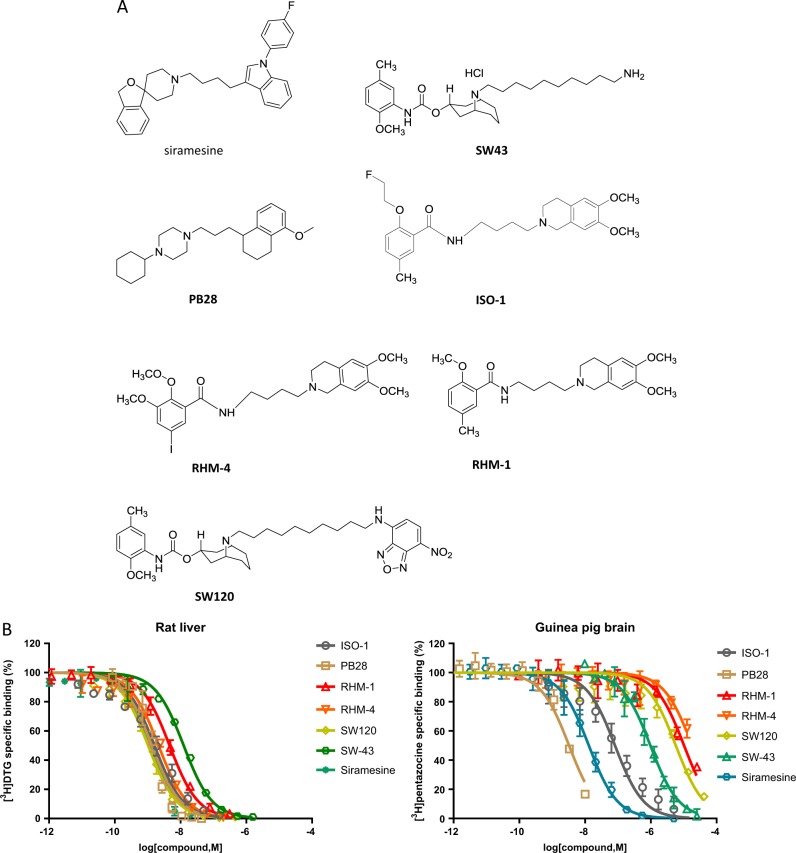
Chemical structures and assessment of binding affinities for sigma-1 and sigma-2 receptors. **a** Chemical structures of sigma-2 ligands used in the current study. **b** Competition curves of [^3^H](+)-pentazocine binding for sigma-1 receptors on guinea pig brain membranes and [^3^H]DTG binding for sigma-2 receptors on rat liver membranes in the presence of increasing concentrations of non-radioactive sigma-2 ligands

**Fig. 2:**
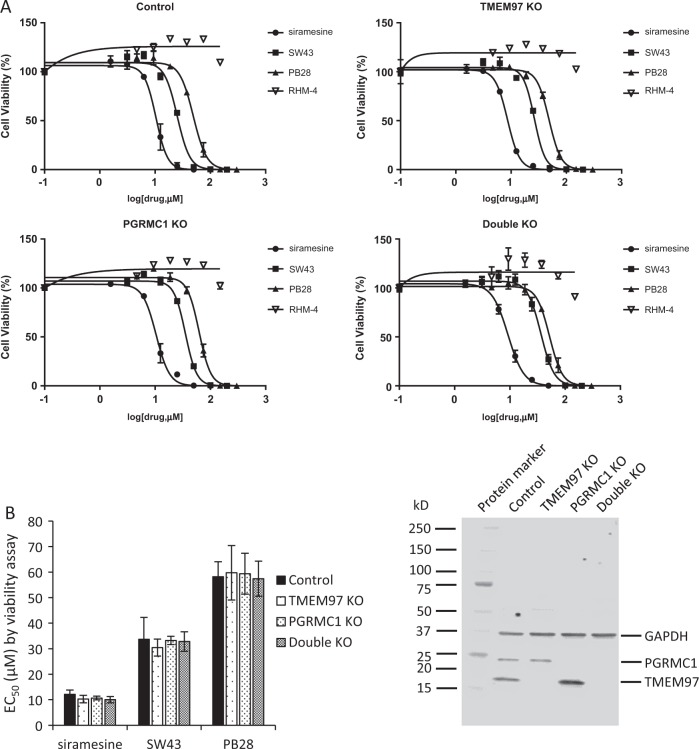
Cytotoxicity of sigma-2 ligands determined by cell viability assay. Control, TMEM97 KO, PGRMC1 KO, or double KO cells were treated with increasing concentrations of sigma-2 ligands for 24 h. The cell viability was then determined by CellTiter Glo® chemiluminescent assay. **a** Representative dose–response curves of cell viability for the control and knockout cell lines. **b** EC_50_ values of sigma-2 ligands were represented as bar graphs. EC_50_ values were reported as mean ± SD from at least two independent experiments performed in triplicates. No significant difference existed for EC_50_ values of sigma-2 ligands in TMEM97 KO, PGRMC1 KO, or double KO cells vs control cells by 2-way Anova analysis. **c** Representative western blot analysis for TMEM97 and PGRMC1 in the control and knockout cell lines used in the current study

Sigma-2 ligands were previously reported as potential therapeutic drugs in various cancer cells in cell culture and in animal models. In order to study whether the cytotoxicity of sigma-2 ligands was mediated by TMEM97 or PGRMC1, we compared cell-killing potency of sigma-2 ligands in control, TMEM97 KO, PGRMC1 KO, and double KO HeLa cells. These cell lines were previously created in our laboratory using CRISPR/Cas9 technology and western blot data demonstrated that TMEM97 or/and PGRMC1 proteins were completely removed in the knockout cell lines^16^. Three sigma-2 agonists **SW43**, siramesine and **PB28**, and three sigma-2 antagonists **RHM-4**, **RHM-1,** and **ISO-1** were studied in this study. The diverse chemical structures of these ligands and their binding affinities obtained by the competition assay for sigma-1 and sigma-2 receptors were shown in Fig. 1 and Table 1. The control and knockout cells were incubated with sigma-2 ligands at increasing concentrations for 24 h and cell viability assay was performed. Dose–response curves were generated as shown in Fig. 2a. EC_50_ values of sigma-2 ligands in each cell line determined from the dose–response curves are shown in Fig. 2b and Table 2. The data showed that knockout of TMEM97, PGRMC1, or both proteins did not affect EC_50_ values of sigma-2 agonists. Similarly, sigma-2 antagonists exhibited little cytotoxicity (EC_50_ > 200 µM) in all the four cell lines as expected (Fig. 2a and Fig. S2A). These data suggest that sigma-2 ligand-induced cytotoxicity is not mediated by TMEM97, PGRMC1 or both of the proteins.

**Table 1:** The binding affinities of sigma-2 ligands for sigma-1 and sigma-2 receptors

	*K*_i_ for sigma-1 receptors (nM) mean ± SD	*K*_i_ for sigma-2 receptors (nM) mean ± SD
Siramesine	3.0 ± 0.5	1.6 ± 0.1
SW43	277.9 ± 65.0	12.9 ± 2.3
PB28	1.1 ± 0.1	1.1 ± 0.3
RHM-4	3606.0 ± 533.3	2.4 ± 0.2
RHM-1	1925.3 ± 625.4	3.8 ± 0.7
ISO-1	23.9 ± 1.9	2.0 ± 0.6
SW120	1942.3 ± 262.3	1.0 ± 0.3

**Table 2:** EC_50_ values of sigma-2 ligands in control, TMEM97 KO, PGRMC1 KO, and double KO cell lines

	Control (µM) mean ± SD	TMEM97 KO (µM) mean ± SD	PGRMC1 KO (µM) mean ± SD	Double KO (µM) mean ± SD
Siramesine	12.1 ± 1.7	10.3 ± 1.49	10.7 ± 0.7	10.0 ± 1.3
SW43	33.7 ± 8.6	30.4 ± 3.4	33.2 ± 1.6	32.8 ± 3.8
PB28	58.2 ± 5.9	59.8 ± 10.7	59.4 ± 8.0	57.4 ± 6.9
RHM-4	>200	>200	>200	>200
RHM-1	>200	>200	>200	>200
ISO-1	>200	>200	>200	>200

The aforementioned TMEM97 KO cell line was generated by CRISPR/Cas9 technology using guide RNA1. We tested whether TMEM97 KO cell lines generated using different guide RNAs resulted in the same results. We performed cell viability assays on TMEM97 KO cell lines generated using guide RNA2 (TMEM97 KO-g2) and guide RNA3 (TMEM97 KO-g3). The data showed that the sigma-2 agonists kill cells with similar EC_50_ values in the control, TMEM97 KO-g2 and TMEM97 KO-g3 cells (Fig. S2B). Moreover, we performed cell viability assay on another set of single protein knockout cell lines, i.e., three clones of PGRMC1 KO cells and three clones of corresponding control cells. The data showed that knockout of PGRMC1 did not affect EC_50_ values of the sigma-2 agonists (Fig. S2C). All cell lines used in the study were subject to western blot analysis to confirm that TMEM97, PGRMC1, or both proteins were stably knocked out throughout the entire study (Fig. 2b and Fig. S2B and S2C). These data further indicated that TMEM97, PGRMC1, or both did not appear to mediate sigma-2 ligand-induced cell death.

**Fig. 3:**
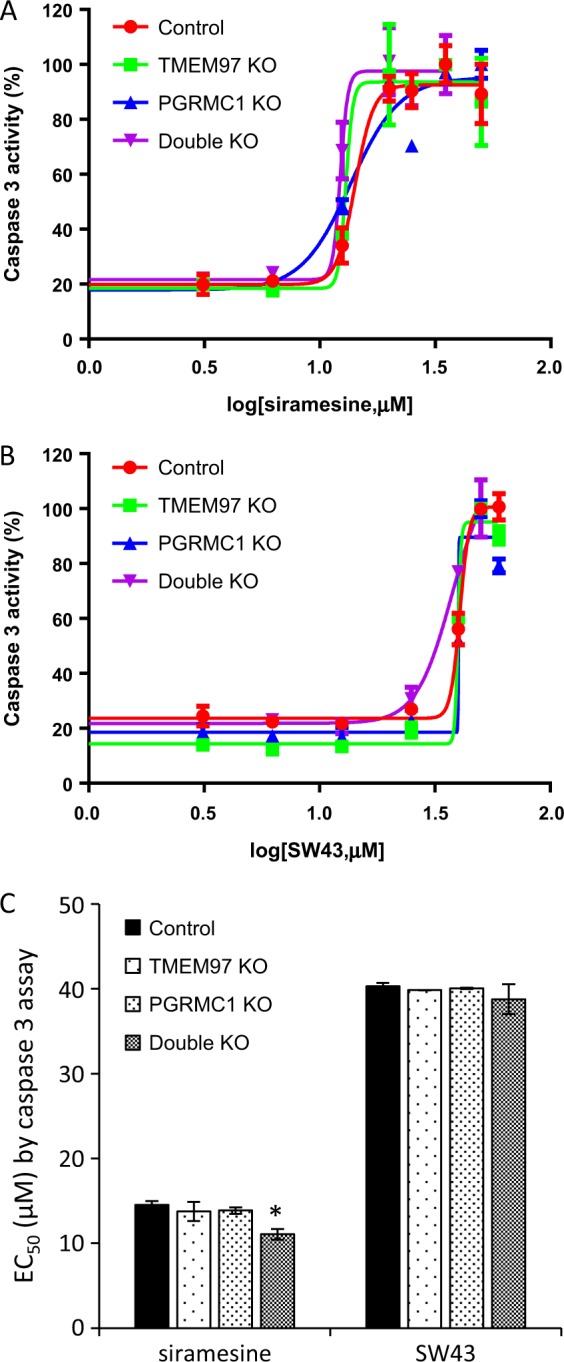
Caspase-3 activities induced by sigma-2 ligands. Control, TMEM97 KO, PGRMC1 KO, or double KO cells were treated with increasing concentrations of sigma-2 ligands for 24 h. Caspase-3 activation was then determined by Caspase-Glo 3/7 Assay. **a**, **b** Representative dose–response curves of caspase-3 activity for siramesine (**a**) and **SW43** (**b**). **c** EC_50_ values of caspase-3 activation of sigma-2 ligands were represented as bar graphs. EC_50_ values were reported as mean ± SD in at least two independent experiments performed in triplicates. No significant difference existed for EC_50_ values of sigma-2 ligands in TMEM97 KO, PGRMC1 KO, or double KO cells vs the control cells by two-way Anova analysis with one exception, i.e., there was a significant difference (asterisk) of EC_50_ of siramesine in double KO cells vs control cells

In order to study whether TMEM97, PGRMC1, or both mediate caspase-3 activation in sigma-2 ligand-induced cell death, control, TMEM97 KO, PGRMC1 KO and double KO cells were treated with siramesine and **SW43** for 24 h and then a caspase-3/7 assay was performed. Dose responsive curves were generated and EC_50_ values were determined (Fig. 3). The data showed that the EC_50_ values for control, TMEM97 KO and PGRMC1 KO cell lines were identical. The EC_50_ values generated by caspase-3/7 assays were comparable to the EC_50_ values determined by the cell viability assays (data not shown). These data suggest that TMEM97 or PGRMC1 did not mediate sigma-2 ligand-induced caspase-3 activation.

### Sigma-1 receptor ligands did not block sigma-2 ligand-induced cytotoxicity

**Fig. 4:**
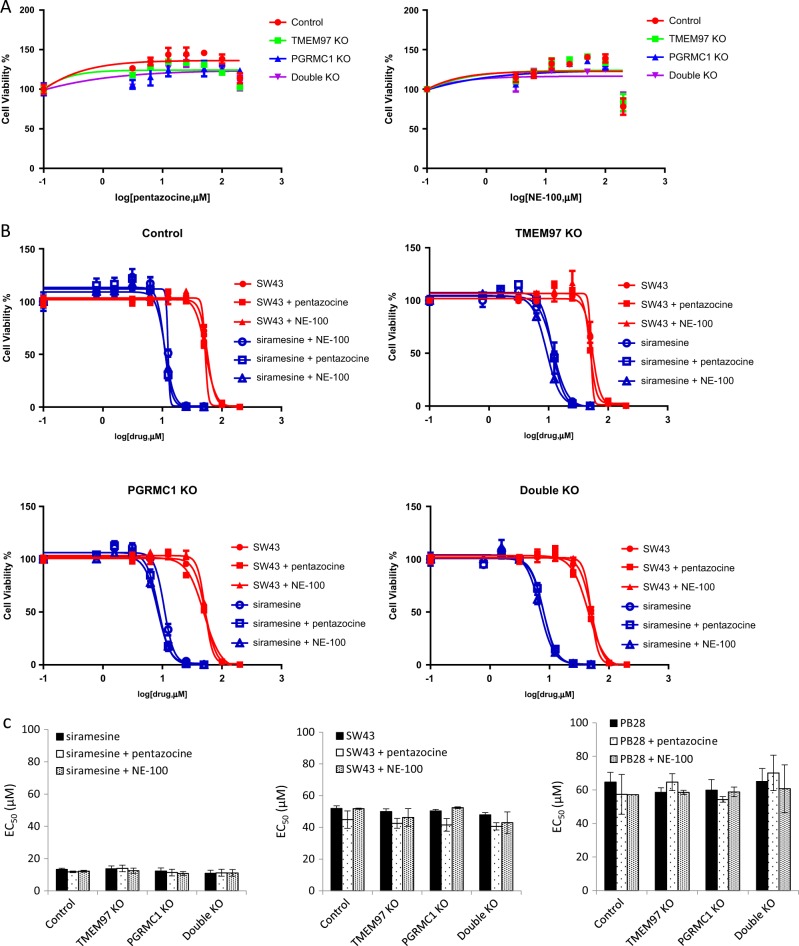
Sigma-1 ligands did not block sigma-2 ligand cytotoxicity. **a** A sigma-1 agonist, (+)-pentazocine, and a sigma-1 antagonist, NE-100, did not exhibit cytotoxicity in the control and knockout cells. **b** Representative dose–response curves of cell viability for siramesine and **SW43** in the absence or presence of 30 μM (+)-pentazocine or NE-100. **c** EC_50_ values of sigma-2 ligands were represented as bar graphs. EC_50_ values were reported as mean ± SD in at least two independent experiments performed in triplicates. No significant difference existed for EC_50_ values of sigma-2 ligands plus (+)-pentazocine or NE-100 vs sigma-2 ligands only in all the four cell lines by two-way Anova analysis

The sigma-2 ligands used in the current study are also expected to bind to sigma-1 receptors in the concentration range used in the cell viability assay (Fig. 1). In order to study whether sigma-2 ligand-induced cytotoxicity is mediated by sigma-1 receptors, we used a sigma-1 agonist, (+)-pentazocine, or sigma-1 antagonist, NE-100, to block the sigma-1 binding site and then examined sigma-2 ligand-induced cytotoxicity. We first showed that (+)-pentazocine or NE-100 by itself did not exhibit cytotoxicity (EC_50_ > 200 µM) in control, TMEM97 KO, PGRMC1 KO, and double KO cells (Fig. 4a). We pre-incubated cells with vehicle (cell culture media), 30 µM (+)-pentazocine, or 30 µM NE-100 for 1 h, then treated cells with sigma-2 agonists in the absence or presence of (+)-pentazocine or NE-100 for 24 h. The cell viability data showed that neither (+)-pentazocine nor NE-100 blocked sigma-2 ligand-induced cytotoxicity (Fig. 4b, c), suggesting that sigma-1 receptors did not mediate the observed sigma-2 ligand-induced cytotoxicity.

### The DTG residual binding site did not appear to mediate cytotoxicity of sigma-2 ligands

**Fig. 5:**
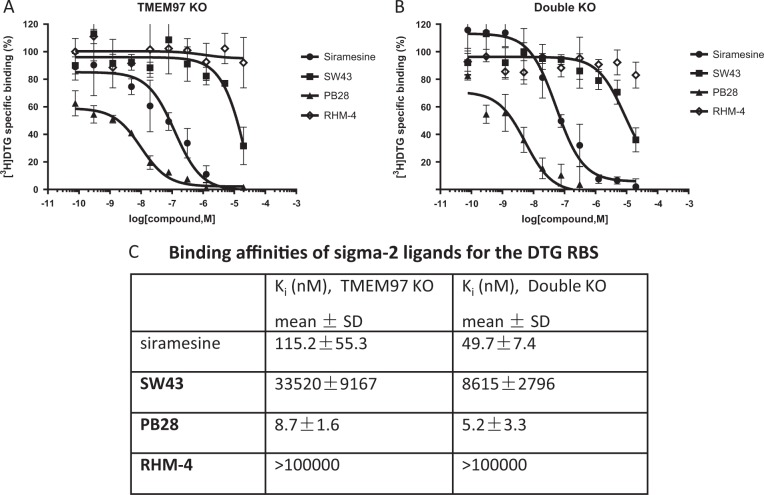
Assessment of binding affinities of sigma-2 ligands for the DTG RBS. **a**, **b** Competition curves of [^3^H]DTG (45 nM) binding on TMEM97 KO (**a**) and double KO (**b**) cell membranes in the presence of increasing concentrations of non-radioactive sigma-2 ligands. 500 nM of (+)-pentazocine was used to mask the sigma-1 receptor binding site. The non-specific binding was determined in the presence of 60 μM cold DTG. **c** The binding affinities (*K*_i_) of sigma-2 ligands for the DTG RBS. *K*_i_ values were reported as mean ± SD in three independent experiments performed in duplicates

Our laboratory recently reported that [^125^I]RHM-4 binding was completely eliminated after TMEM97 was knocked out, suggesting that [^125^I]RHM-4 had one specific binding site, the sigma-2 receptor/TMEM97. We also observed that [^3^H]DTG binding was significantly but not completely reduced after TMEM97 was knocked out, suggesting that [^3^H]DTG bound to TMEM97 as well as one or more unidentified proteins. We called the remaining DTG binding site “the DTG residual binding site (RBS)”. We then explored whether the DTG RBS mediates the cytotoxicity of sigma-2 ligands used in this study. We determined the inhibition constant (*K*_i_) values of sigma-2 ligands for the DTG RBS using a competition assay (Fig. 5a-c). The data showed that the *K*_i_ values for **SW43** was 33,520 or 8615 nM on TMEM97 KO or double KO cell membranes, respectively, suggesting that **SW43** had little binding affinity for the DTG RBS, thus the cytotoxicity of **SW43** on TMEM97 KO cells (EC_50_ = 30.4 μM) or double KO cells (EC_50_ = 32.8 μM) did not appear to be mediated by the DTG RBS. Moreover, we compared the *K*_i_ values and EC_50_ values for siramesine and **PB28**. As shown in Fig. 5c, the *K*_i_ values of siramesine were 115.2 or 49.7 nM on TMEM97 KO or double KO cell membranes, respectively, and the *K*_i_ values for **PB28** were 8.7 or 5.2 nM on TMEM97 KO or double KO cell membranes, respectively. As shown in Table 2, the EC_50_ values of siramesine were 10.3 or 10 μM for TMEM97 KO or double KO cells, respectively, and the EC_50_ values of **PB28** were 59.8 or 57.4 μM for TMEM97 KO or double KO cells, respectively. These data indicated that the *K*_i_ values of siramesine and **PB28** negatively correlated with their EC_50_ values. These data do not exclude the possibility that the DTG RBS mediates the cytotoxicity of siramesine and **PB28**. However, the data showed that the DTG RBS was not fully responsible for siramesine and **PB28** cytotoxicity.

### Knockout of TMEM97, PGRMC1, or both proteins reduced the initial internalization rate of the sigma-2 fluorescent ligand, SW120

**Fig. 6:**
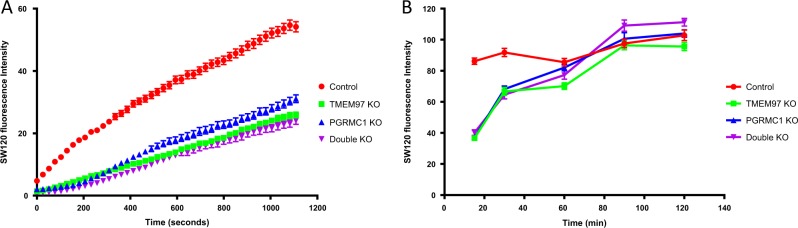
Internalization kinetics of SW120. **a** Control, TMEM97 KO, PGRMC1 KO, or double KO cells were incubated with 100 nM **SW120** at room temperature and time-lapse images were immediately taken after addition of **SW120** at 25-s intervals for 18 min using an inverted confocal microscope. Fluorescence intensity of **SW120** in the cells was quantified and presented as function of internalization time. **b** Cells were incubated with 100 nM **SW120** for 15, 30, 60, 90, and 120 min at room temperature. Confocal images were taken at indicated time points. Fluorescence intensity of **SW120** in the cells was quantified and presented as function of internalization time. The fluorescence intensities were reported as mean ± SEM in three independent experiments

In order to determine whether TMEM97, PGRMC1, or both proteins together are responsible for internalization of sigma-2 ligands, the internalization rate of a sigma-2 fluorescent ligand, **SW120**, was measured. Control, TMEM97 KO, PGRMC1 KO, or double KO cells were incubated with 100 nM **SW120** and time-lapse images were immediately taken after addition of **SW120** at 25-sec intervals for 18 min using an inverted confocal microscope (Fig. 6a and Fig. S3). Compared to the control cells, TMEM97 KO, PGRMC1 KO and double KO cells exhibited reduced internalization rates of **SW120**. The data suggested that **SW120** internalization was in part mediated by TMEM97 and/or PGRMC1. On the other hand, the observation that **SW120** could still enter TMEM97 KO, PGRMC1 KO, and double KO cells suggested that **SW120** uptake could also be mediated in a TMEM97-independent and/or PGRMC1-independent manner. We also examined **SW120** uptake at longer time points, i.e., 15, 30, 60, 90, and 120 min. As shown in Fig. 6b, although **SW120** uptake signals at 15 and 30 min time points were lower in the knockout cells than the control cells, **SW120** uptake signals at 60, 90, and 120 min were essentially the same for all the cell lines. These results are consistent with our data that sigma-2 ligands did not show any difference in cytotoxicity after 24 h treatment in the control and knockout cells.

## Discussion

In this study, we showed that the knockout of TMEM97, PGRMC1, or both proteins in HeLa cells did not affect the EC_50_ values of sigma-2 ligands, suggesting that sigma-2 ligand-induced cytotoxicity is not mediated by TMEM97, PGRMC1, or the combination of both proteins. Furthermore, we determined the *K*_i_ values of sigma-2 ligands for the DTG RBS and the data suggest that the DTG RBS is not, at least, fully responsible for sigma-2 ligand-induced cytotoxicity.

In order to study whether the sigma-2 receptor mediates sigma-2 ligand-induced cell death, we treated control, TMEM97 KO, PGRMC1 KO, or double KO cells with various sigma-2 ligands developed by our laboratory as well as by other groups. Our data showed that sigma-2 ligands exhibited cytotoxicity with essentially the same potency in the control and the knockout cells (Figs. 2 and 3, and Fig. S2). The data suggest that the cell-killing effects of sigma-2 ligands were not mediated by TMEM97, PGRMC1, or the combination of both proteins. Since the sigma-2 ligands also possess nanomolar to micromolar binding affinities to sigma-1 receptors, we examined whether sigma-2 ligand-induced cytotoxicity was mediated by sigma-1 receptors. Our data showed that a sigma-1 agonist, pentazocine, and an antagonist, NE-100, did not block sigma-2 ligand-induced cytotoxicity (Fig. 4a-c). Also, the EC_50_ values of sigma-2 ligands did not appear to correlate with their *K*_i_ values for sigma-1 receptors (Tables 1 and 2). These results suggest that sigma-2 ligand-induced cytotoxicity was not mediated by sigma-1 receptors. Future studies using sigma-1 receptor knockout cell lines might help further delineate this observation.

Our group recently found that [^3^H]DTG binding was significantly reduced, but not completely abolished, in the TMEM97 KO and double KO cells. This observation suggests that there exists an additional DTG binding site, a site we have termed the “DTG residual binding site (RBS)”. The *K*_d_ for the [^3^H]DTG RBS are 302.0 and 402.6 nM for TMEM97 KO and double KO cell membranes^16^, respectively, indicating that [^3^H]DTG binding affinity to this residual site is relatively low when compared to its binding affinity for TMEM97 proteins in the control cell membranes (*K*_d_ = 14.8 nM). The *B*_max_ for the DTG RBS are 1218 and 1374 fmole/mg for TMEM97 KO and double KO cell membranes, respectively, indicating that the maximal binding capacity is moderate when compared to the *B*_max_ (2664 fmole/mg) for control cell membranes. The DTG RBS is obviously not TMEM97 or PGRMC1 because this residual site exists in the double knockout cells. The DTG RBS is not traditional sigma-1 receptor binding site since 500 nM (+)-pentazocine could not block [^3^H]DTG binding to this residual site. We also examined whether the DTG RBS is responsible for sigma-2 ligand-induced cytotoxicity. We performed the competition assays and determined *K*_i_ values of sigma-2 ligands for the DTG RBS (Fig. 5). Our data showed that **SW43** has a low affinity (33,520 or 8615 nM on TMEM97 KO or double KO cell membranes, respectively) for this residual binding site. Thus it is unlikely that the DTG RBS mediates **SW43** cytotoxicity. Our data also showed that *K*_i_ values for siramesine and **PB28** are in the nanomolar range (i.e., 49.7 and 5.2 nM, respectively, on double KO cell membranes), suggesting that these two sigma-2 ligands possess good binding affinities to the DTG RBS. However, their EC_50_ values were in the micromolar range (i.e., 10.0 and 57.4 μM for double KO cell membranes) in the cytotoxicity assay (Table 2). The fact that EC_50_ values of siramesine and **PB28** are ~200 and ~1000 fold higher, respectively, than their *K*_i_ values again raises the question whether the DTG RBS mediates the cytotoxicity of siramesine and **PB28**. Particularly, there is no correlation between the *K*_i_ and EC_50_ values for siramesine and **PB28**. Thus, although these data do not exclude the possibility that the DTG RBS may mediate the cytotoxicity of certain sigma-2 ligands, the DTG RBS is not, at least, fully responsible for sigma-2 ligand-induced cell death, and other mechanisms may be involved in sigma-2 ligand-induced cytotoxicity.

It is reported that siramesine, **SW43** and **PB28** all accumulated in lysosomes and increased lysosomal pH^26–28^. Siramesine treatment decreased lysosomal cysteine cathepsin activity and decreased lysosomal stability for hypotonic conditions. Siramesine, **SW43** and **PB28** also induced reactive oxygen species (ROS). Therefore, these results suggest that sigma-2 ligand-induced lysosomal dysfunction and ROS production are early events that may be responsible for sigma-2 ligand-induced cytotoxicity. Whether TMEM97, PGRMC1, or the DTG RBS mediate sigma-2 ligand-induced lysosome dysfunction and ROS production needs to be studied.

Our data showed that knockout of TMEM97, PGRMC1, or both proteins reduced the internalization rate of a sigma-2 ligand, **SW120** (Fig. 6a). These data are consistent with our prior report that the internalization of LDL is mediated by the LDLR/TMEM97/PGRMC1 complex since knocking out either TMEM97, PGRMC1, or both proteins dramatically reduced the rate of uptake of radiolabeled LDL. Whether or not the LDLR/TMEM97/PGRMC1 complex mediates the internalization of sigma-2 ligands is not known and deserves further investigation. It is also important to note that although the concentrations of **SW120** in TMEM97 KO and PGRMC1 KO cells were lower than that in control cells at early time points of internalization, the cellular concentrations of **SW120** eventually reach the same plateau in the knockout and control cells at late time points (>60 min) (Fig. 6b). Thus the final concentrations of sigma-2 ligands inside cells under the conditions of the cell viability and caspase-3/7 assays are identical in both the control and knockout cells, and this could explain why the initial sigma-2 ligand internalization rate difference did not affect EC_50_ values in the knockout cells versus control cells.

In summary, we have provided evidence that sigma-2 receptor/TMEM97 and PGRMC1 do not mediate the cytotoxicity of a panel of sigma-2 ligands. Our work with the TMEM97 and double knockout cells should facilitate elucidating the mechanisms of sigma-2 ligand cytotoxicity.

## Materials and methods

### Cell culture

HeLa human cervical cancer cells were from American Type Culture Collection (ATCC, Manassas, VA). Control, TMEM97 KO, PGRMC1 KO and TMEM97/PGRMC1 double KO HeLa cell lines were grown in MEM containing 10% fetal bovine serum, 2 mM l-glutamine, 1% non-essential amino acids, 100 units/ml penicillin, and 100 μg/ml streptomycin. All the cell lines were maintained at 37 °C in a humidified incubator with a 5% CO_2_/95% air atmosphere.

### Western blot analysis

Cells were lysed in radioimmunoprecipitation assay (RIPA) buffer (Sigma-Aldrich, St. Louis, MO, USA) containing 150 mM NaCl, 1.0% IGEPAL^®^ CA-630, 0.5% sodium deoxycholate, 0.1% SDS, 50 mM Tris, pH 8.0 supplemented with protease inhibitor cocktail, and phosphatase inhibitor cocktail 2 and 3 (Sigma-Aldrich, St. Louis, MO, USA). The cells were sonicated briefly, centrifuged at 13,000×*g* for 20 min at 4 °C, and the supernatant collected. The protein concentration was determined using a Bio-Rad Dc protein assay kit (Bio-Rad Laboratories, Hercules, CA, USA). Lysates containing 20 µg of protein were run on a 4–20% acrylamide gel and transferred to a PVDF membrane using the Trans-Blot Turbo Transfer System (Bio-Rad Laboratories, Hercules, CA, USA). The PVDF membrane was incubated with Odyssey blocking buffer (Licor Biotechnology, Lincoln, NE) for 1 h at room temperature, then overnight with a rabbit anti-TMEM97 antibody (Aviva Systems Biology, San Diego, CA) at a 1:8000 dilution, or a rabbit anti-PGRMC1 antibody (Sigma-Aldrich) at a 1:1000 dilution at 4 °C, and finally with the secondary antibody, IRDye 800CW anti-rabbit IgG (Licor Biotechnology) at a 1:15,000 dilution. The signals were detected and quantified using the Odyssey® CLx Infrared Imaging System (Licor Biotechnology).

### Cell viability assay

The cytotoxicity of compounds was determined using the CellTiter Glo® chemiluminescent assay (Promega, Madison, WI) that measures ATP. Cells were plated in black wall clear bottom 96-well plates at 5000 cells per well 24 h before treatment. Each compound was dissolved in DMSO and serially diluted in culture medium to acquire the desired concentrations. The final concentration of DMSO in the cell culture medium was no more than 1.0%. After 24 h treatment with the various compounds, 25 μl of the CellTiter Glo® Solution Reagent was added to each well. Then plates were immediately read on a Perkin Elmer Enspire® Multimode Plate Reader. Luminescence detected for each well was normalized to the untreated control and data was calculated as percent viability. The EC_50_, defined as the concentration of the sigma ligand required to inhibit cell proliferation by 50% relative to untreated cells, was determined from the dose–response curves generated using GraphPad Prism version 6 (GraphPad Software, Inc. La Jolla, CA).

### Caspase-3 assay

HeLa cells were plated in white opaque 96-well plate at a cell density of 5000 cells per well for 24 h. The cells were then treated with sigma-2 ligands, **SW43**, and siramesine at increasing concentrations for 24 h. Caspase-3/7 activity was measured by using the Caspase-Glo 3/7 Assay (Promega).

### Sigma-1 and sigma-2 receptor binding assays

The sigma-1 and sigma-2 receptor binding affinities of sigma-2 ligands were determined using a competition assay as previously described^32,33^. For the sigma-1 binding assay, 100 μg guinea pig brain membrane homogenates and non-radioactive compounds were incubated with 5 nM [^3^H](+)-pentazocine in 50 mM Tris–HCl, pH 8.0, and 0.1% bovine serum albumin (BSA) for 90 min at 37 °C. The concentrations of each non-radioactive compound ranged from 0.1 to 10 μM. The non-specific binding was determined in the presence of 10 μM haloperidol. For the sigma-2 binding assay, 60 μg Sprague Dawley rat liver membrane homogenates and non-radioactive compounds were incubated with 5 nM [^3^H]DTG in 50 mM Tris–HCl, pH 8.0, and 0.1% BSA for 60 min at 37 °C. The concentrations of each non-radioactive compound ranged from 0.1 nM to 10 μM. 500 nM of (+)-pentazocine was added to mask the sigma-1 receptor binding site. The non-specific binding was determined in the presence 10 μM DTG.

The binding affinities of sigma-2 ligands for the DTG RBS were determined using a competition assay as previously described^13,33^ with minor modification. For cell harvesting, TMEM97 KO or TMEM97/PGRMC1 double KO HeLa cells were scraped from culture dishes in ice-cold Phosphate-Buffered Saline (PBS) and collected by centrifugation. The cell pellets were immediately stored at −80 °C until use. For cell membrane homogenate preparation, the cell pellets were re-suspended in 10 mL ice-cold PBS, and homogenized using Wheaton overhead stirrer (120 Vac Overhead Stirrer, Millville, NJ, USA) at the speed of 2 for 30 sec. The cell homogenates were then centrifuged for 20 min at 31,000×*g* at 4 °C. After centrifugation, the supernatant was discarded and the pellets were re-suspended in 1 mL ice-cold PBS and stored at −80 °C freezer until use. For the DTG RBS binding assay, 100 μg TMEM97 KO or TMEM97/PGRMC1 double KO HeLa cell membrane homogenates and non-radioactive compounds with concentrations ranging from 1 nM to 10 μM were incubated with 45 nM [^3^H]DTG in 50 mM Tris–HCl, pH 8.0, and 0.1% BSA for 60 min at 37 °C. 500 nM (+)-pentazocine was added to mask the sigma-1 receptor binding site. The non-specific binding was determined in the presence of 60 μM cold DTG.

After incubation, the bound ligands were filtrated with a M-24 Brandel filtration system (Brandel, Gaithersburg, MD, USA), collected on glass fiber papers (Whatman grade 934-AH, GE Healthcare Bio-Sciences, Pittsburgh, PA), and counted with a MicroBeta2 Microplate counter 2450 (Perkin Elmer, Boston, MA). Inhibition constant (*K*_i_) values of tested compounds were determined by using the Cheng and Prussoff equation^34^:

$$K_{\mathrm{i}} = \frac{{{\mathrm{IC}}_{50}}}{{1 + L_{\mathrm{t}}/K_{\mathrm{d}}}}$$

where *K*_d_ values of [^3^H](+)-pentazocine on guinea pig brain membrane homogenates, [^3^H]DTG on rat liver membrane homogenates, [^3^H]DTG on TMEM97 KO or TMEM97/PGRMC1 double KO HeLa cell membrane homogenates were 1.5, 20.7 nM (Fig. S1), 302, and 402.6 nM^16^, respectively, and *L*_t_ is the concentration of radioligand used in each assay. *K*_i_ values were reported as mean ± SD from at least three independent experiments.

### Internalization of SW120

HeLa cells were plated on 35-mm-diameter glass-bottom dishes at 2 × 10^5^ cells per dish and incubated for 24 h. Prior to imaging, media was removed and fresh media containing 100 nM **SW120** was added to the dishes. **SW120** uptake in the live cells at room temperature was imaged using an inverted confocal microscope (Leica STED 3× super-resolution microscope). Images were acquired at 25 s intervals using a ×10 objective lens (using an excitation wavelength of 488 nm, and a 505 to 540 nm bandpass filter). Five optical slices at 2-μm intervals were scanned using the Z-stack function of the Leica imaging software. The brightest optical slice out of the five *Z*-stack optical slices was chosen for fluorescent intensity analysis. Twenty cells were analyzed in each dish using Fiji, an open-source platform based on ImageJ^35^. The fluorescent intensity was calculated as an average of 60 cells from three independent experiments (mean ± SEM).

### Statistical analysis

The results are expressed as the mean ± SD (standard deviation) or mean ± SEM (standard error) from at least three independent experiments performed in duplicate or triplicate. Differences among groups were analyzed by one-way or two-way ANOVA with a Dunnett’s multiple comparisons test using GraphPad Prism version 6 (GraphPad Software, Inc. La Jolla, CA). The significance level is 0.05.

## Supplementary information

Supplemental Material
