# Supplementary material for: TMEM97 and PGRMC1 do not mediate sigma-2 ligand-induced cell death
Source: Cell Death Discov. 2019 Jan 28;5:58. doi: 10.1038/s41420-019-0141-2 (PMC6349905; doi:10.1038/s41420-019-0141-2)
Supplement: Supplementary file 1 — Supplemental Material [file 41420_2019_141_MOESM1_ESM.pdf]

Fig. S1

A

B

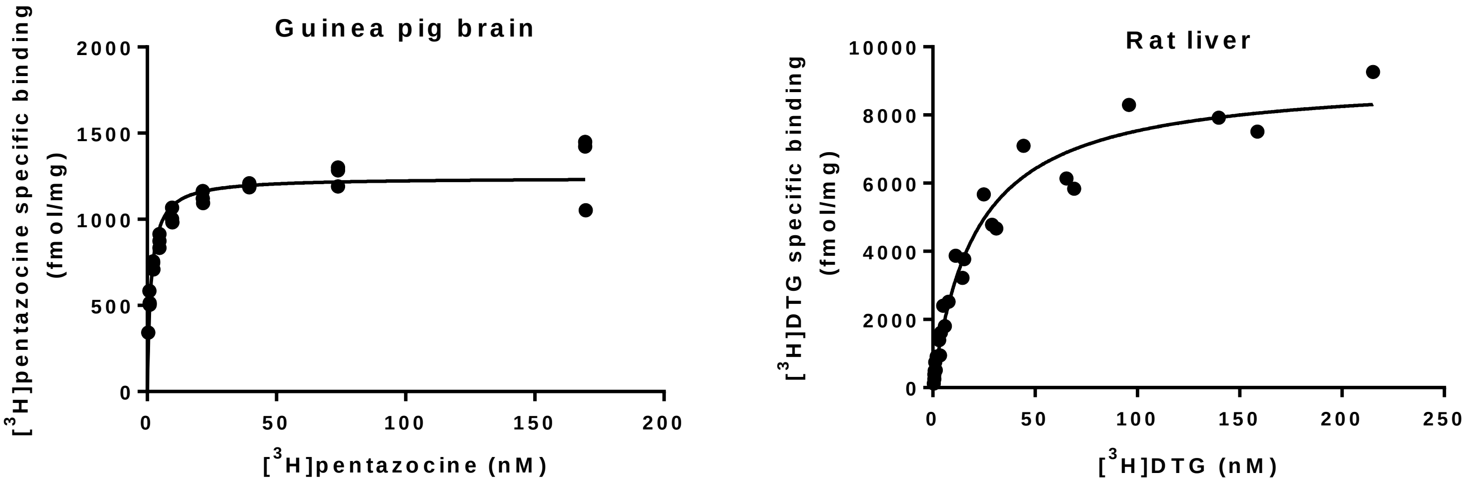

K_d = 1.5 ± 0.2 nM
B_{max} = 1242.0 ± 77.9 fmol/mg

K_d = 20.7 ± 5.2 nM
B_{max} = 8958.0 ± 924.9 fmol/mg

Fig. S1. Saturation binding of [^{3}H](+)-pentazocine for sigma-1 receptors on guinea pig brain membranes and [^{3}H]DTG for sigma-2 receptors on rat liver membranes. The concentrations of [^{3}H](+)-pentazocine ranged from 0.6 to 175 nM, and the concentrations of [^{3}H]DTG ranged from 0.9 to 225 nM. For [^{3}H](+)-pentazocine binding, the nonspecific binding was determined in the presence of 10 µM haloperidol. For [^{3}H]DTG binding, 500 nM (+)-pentazocine was added to mask the sigma-1 receptor binding site and the nonspecific binding was determined in the presence 10 µM DTG. The K_d and B_{max} values were determined by a nonlinear regression analysis using GraphPad Prism software. K_d and B_{max} of [^{3}H](+)-pentazocine were 1.5 ± 0.2 nM and 1242.0 ± 77.9 fmol/mg, respectively. K_d and B_{max} of [^{3}H] DTG were 20.7 ± 5.2 nM and 8958.0 ± 924.9 fmol/mg, respectively. The K_d and B_{max} values were reported as mean ± SD in at least three independent experiments performed in duplicates. The K_d values of [^{3}H](+)-pentazocine or [^{3}H]DTG were used to calculate K_i values of compounds in the current study for sigma-1 receptors or sigma-2 receptors, respectively.

Fig. S2A

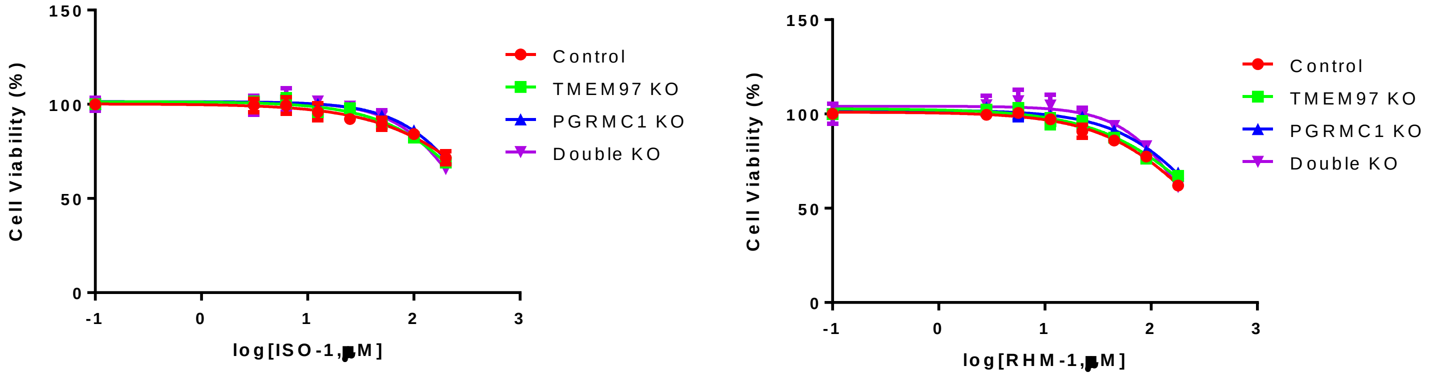

Fig. S2B

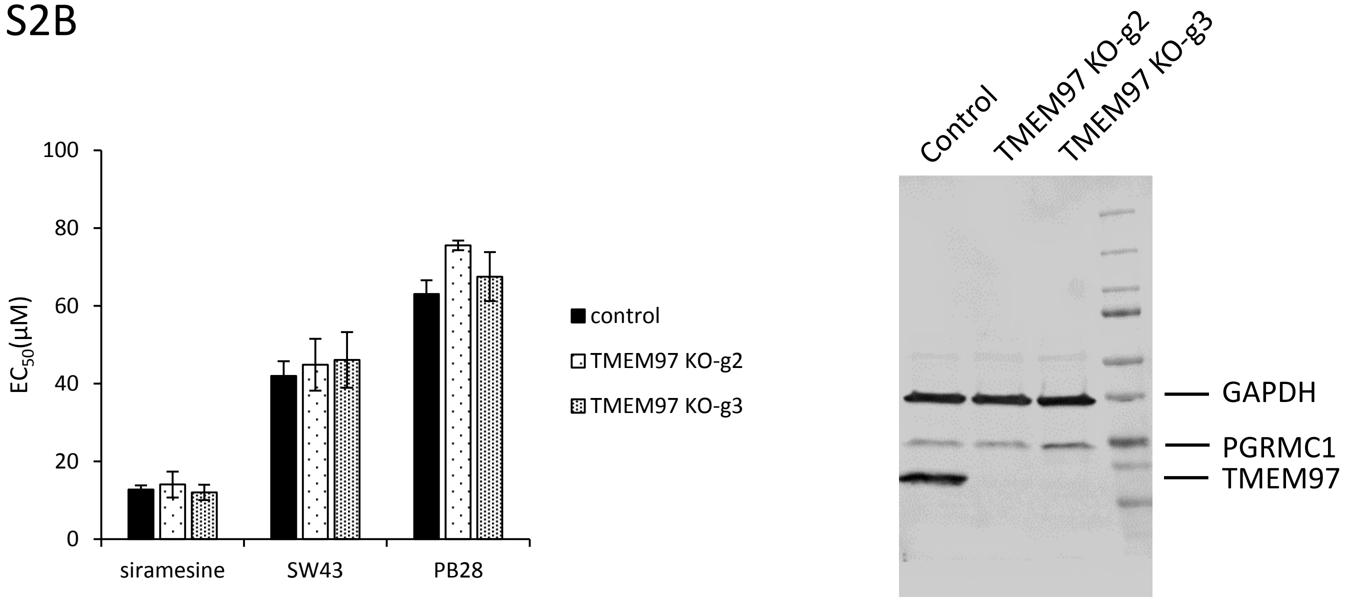

Fig. S2C

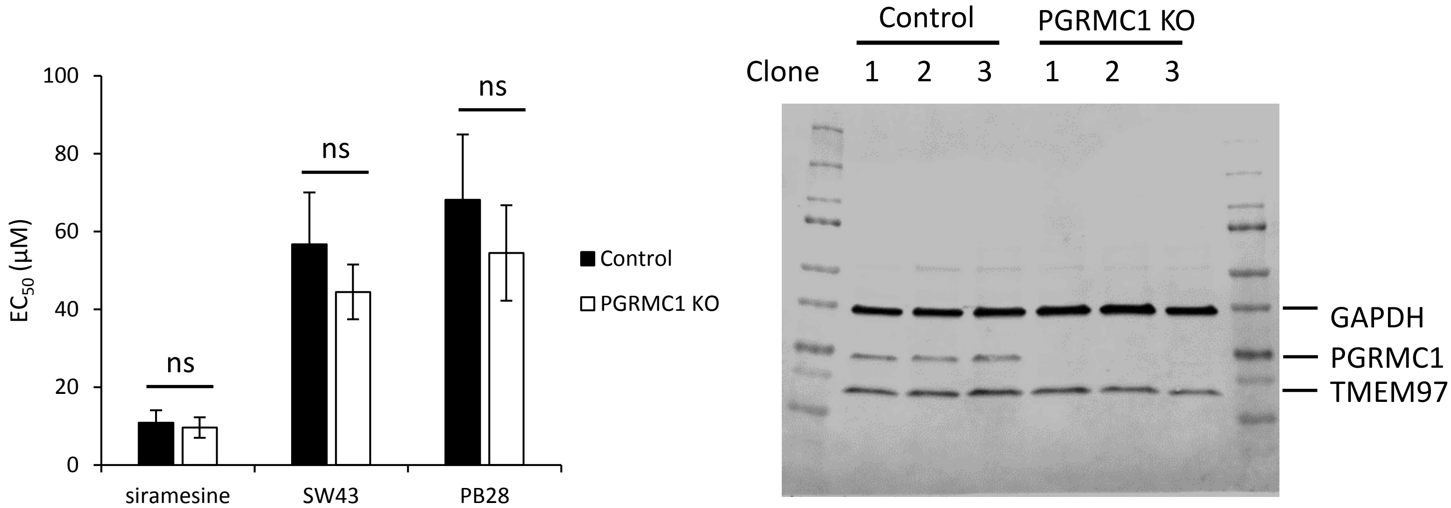

Fig.S2. Cell viability assay to assess cytotoxicity of sigma-2 ligands in the TMEM97 KO or/and PGRMC1 knockout cells and the corresponding control cells. (A) Sigma-2 antagonists, ISO-1 and RHM-1, did not exhibit cytotoxicity to control, TMEM97 KO, PGRMC1 KO and double KO HeLa cells. (B) Cell viability assay was performed on TMEM97 KO cell lines generated using guide RNA2 (TMEM97 KO-g2) and guide RNA3 (TMEM97 KO-g3). The left panel showed that the sigma-2 agonists killed cells with similar EC50 values in control, TMEM97 KO-g2 and TMEM97 KO-g3 cells. The right panel is a western blot confirming that TMEM97 was knocked out in the cell lines throughout the current study. (C) Cell viability assay was performed on three clones of PGRMC1 KO cells and three clones of corresponding control cells. The left panel showed that knockout of PGRMC1 did not affect EC50 values of the sigma-2 agonists. The right panel is a western blot confirming that PGRMC1 was knocked out in the cell lines throughout the current study.

Fig. S3

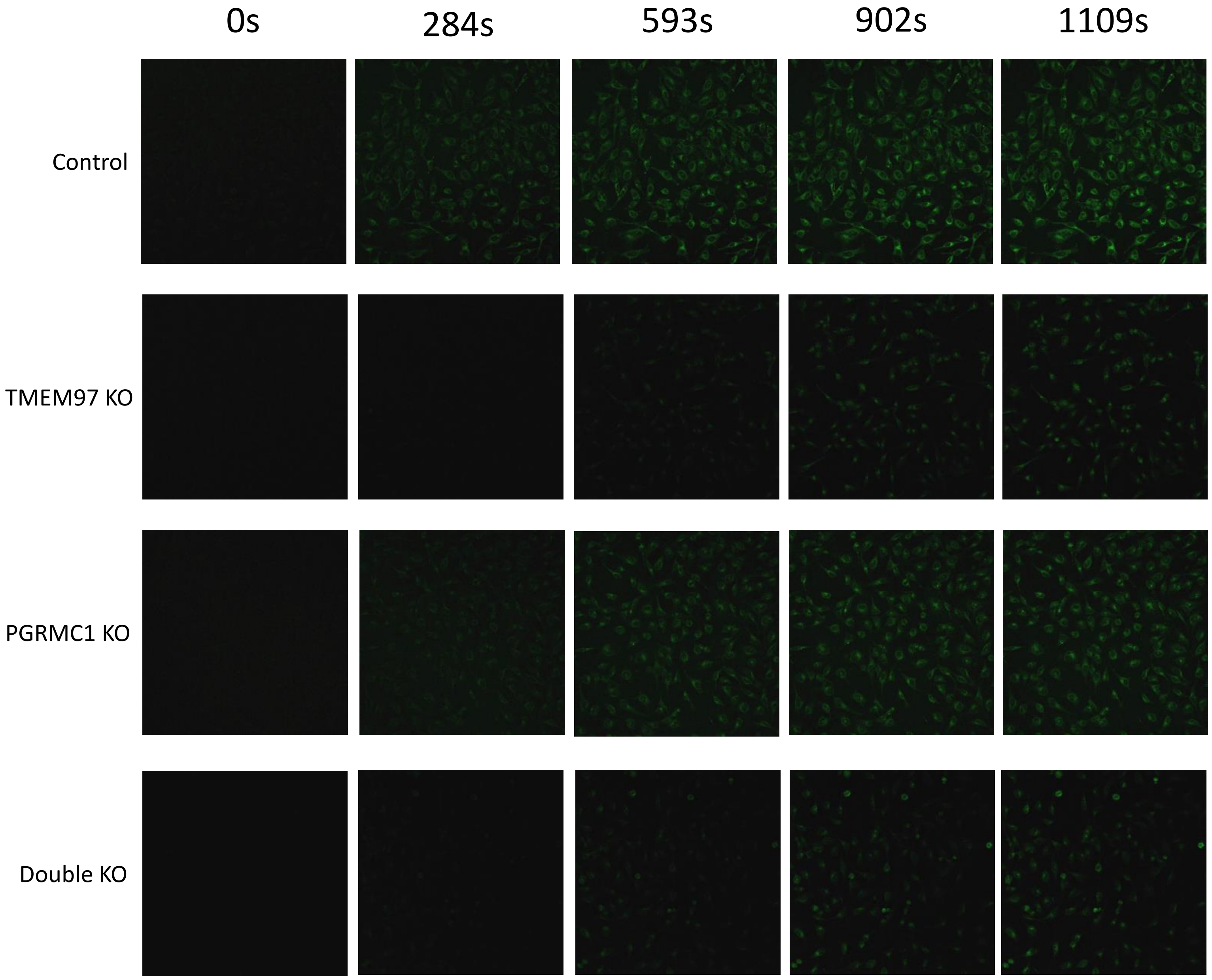

Fig. S3. Real time imaging of **SW120** internalization. Control, TMEM97 KO, PGRMC1 KO or double KO cells were incubated with 100 nM **SW120** and time-lapse images were immediately taken after addition of **SW120** at 25-sec intervals for 18 min using an inverted confocal microscope. Compared to control cells, TMEM97 KO, PGRMC1 KO or double KO cells exhibited reduced internalization rate of **SW120**.
